# Plasticity of neuronal dynamics in the lateral habenula for cue-punishment associative learning

**DOI:** 10.1038/s41380-023-02155-3

**Published:** 2023-07-06

**Authors:** Mauro Congiu, Sarah Mondoloni, Ioannis S. Zouridis, Lisa Schmors, Salvatore Lecca, Arnaud L. Lalive, Kyllian Ginggen, Fei Deng, Philipp Berens, Rosa Chiara Paolicelli, Yulong Li, Andrea Burgalossi, Manuel Mameli

**Affiliations:** 1 The Department of Fundamental Neuroscience, The University of Lausanne, 1005 Lausanne, Switzerland; 2 Institute of Neurobiology and Werner Reichardt Centre for Integrative Neuroscience (CIN), University of Tübingen, 72076 Tübingen, Germany; 3 Graduate Training Centre of Neuroscience, International Max Planck Research School (IMPRS), University of Tübingen, Tübingen, Germany; 4 Institute for Ophthalmic Research, University of Tübingen, Tübingen, Germany; 5 Hertie Institute for AI in Brain Health, University of Tübingen, Tübingen, Germany; 6 The Department of Biomedical Sciences, The University of Lausanne, 1005 Lausanne, Switzerland; 7 School of Life Sciences, Peking University, Beijing, 100871 China; 8 Tübingen AI Center, University of Tübingen, Tübingen, Germany; 9 Inserm, UMR-S 839, 75005 Paris, France

**Keywords:** Neuroscience, Depression

## Abstract

The brain’s ability to associate threats with external stimuli is vital to execute essential behaviours including avoidance. Disruption of this process contributes instead to the emergence of pathological traits which are common in addiction and depression. However, the mechanisms and neural dynamics at the single-cell resolution underlying the encoding of associative learning remain elusive. Here, employing a Pavlovian discrimination task in mice we investigate how neuronal populations in the lateral habenula (LHb), a subcortical nucleus whose excitation underlies negative affect, encode the association between conditioned stimuli and a punishment (unconditioned stimulus). Large population single-unit recordings in the LHb reveal both excitatory and inhibitory responses to aversive stimuli. Additionally, local optical inhibition prevents the formation of cue discrimination during associative learning, demonstrating a critical role of LHb activity in this process. Accordingly, longitudinal in vivo two-photon imaging tracking LHb calcium neuronal dynamics during conditioning reveals an upward or downward shift of individual neurons’ CS-evoked responses. While recordings in acute slices indicate strengthening of synaptic excitation after conditioning, support vector machine algorithms suggest that postsynaptic dynamics to punishment-predictive cues represent behavioral cue discrimination. To examine the presynaptic signaling in LHb participating in learning we monitored neurotransmitter dynamics with genetically-encoded indicators in behaving mice. While glutamate, GABA, and serotonin release in LHb remain stable across associative learning, we observe enhanced acetylcholine signaling developing throughout conditioning. In summary, converging presynaptic and postsynaptic mechanisms in the LHb underlie the transformation of neutral cues in valued signals supporting cue discrimination during learning.

## Introduction

Pavlovian conditioning represents a temporally trackable brain function in which sensory cues are associated with incentive stimuli enabling individuals to anticipate upcoming threats and rewards. This process is at the basis of complex behavioural outcomes including defensive responses such as freezing or avoidance [1–4]. During Pavlovian conditioning, a sensory cue, the conditioned stimulus (CS+), is associated with an unconditioned stimulus (US), an airpuff directed to the eye for instance [4]. Upon re-exposure to the CS+ only, an eyeblink is elicited in the prediction of the subsequent airpuff, indicative of associative learning and the establishment of anticipatory behaviour [1, 4, 5].

Past work found lateral habenula (LHb) neurons with potentiated synaptic glutamate transmission after learning along with CS-mediated phasic excitation of the LHb [6, 7]. Notably, this punishment-predictive cue-mediated LHb neuronal excitation is conserved across species as it is present in humans, non-human primates, rodents, and fish [5, 8–10]. The LHb receives negative-related information from a subcortical network including hypothalamic, limbic, and basal ganglia nuclei [11]. The neural activity within the LHb represents punishments and negative affective states at both sub-second and slower timescales [12]. While LHb neurons phasically increase their activity in response to punishments, persistent synaptic adaptations and enhanced neuronal activity emerge during elevated stress conditions [5, 13–17]. Altogether, these observations support a causal link between the excitation of LHb and the encoding of negative valence and affect [12]. Recent advances pinpoint, however, a degree of diversity in the LHb that is based on both molecular and functional features [18–21]. This suggests that excitation across all LHb cells seems unlikely to be the sole mechanism to support cue-punishment associative learning. Independent and differentially adaptive neuronal populations allow learning and memory storage [22], yet whether this applies to habenula and through which mechanisms remain unknown.

Here, we combined temporally- and spatially-controlled analysis of both neuronal and neurotransmitter dynamics together with an aversive Pavlovian conditioning task in awake rodents to study LHb contribution to learning. We observed that punishments and punishment-predictive cues recruit both pre-and postsynaptic adaptations that enable discrete cell ensembles in the LHb to encode punishment-related associative memories.

## Materials and methods

### Experimental subjects

C57BL/6 J wild-type male mice 7–25 weeks old were used for this study (Janvier lab, France; Charles River, Sulzfeld, Germany). Mice were housed in three to five per cage with water and food ad libitum on a 12:12 h light cycle (lights on at 7 a.m.) in individually ventilated cages (ICV, Innovive, France). All experimental procedures were approved by local authorities and were performed according to guidelines of the respective local ethics committee: the canton of Vaud Cantonal Veterinary Office Committee for Animal Experimentation (Switzerland; License VD3171), in compliance with the Swiss National Institutional Guidelines on animal experimentation; the Regierungspräsidium (Tübingen, Baden-Württemberg, Germany; License CIN07/19G and CIN03/20G) in compliance with the German Animal Welfare Act (TierSchG) and the Animal Welfare Laboratory Animal Ordinance (TierSchVersV).

### Viruses

rAAV-DJ/8/2-hSyn1-eGFP-WPRE (titer: 9.4x10E12 vg/ml), rAAV-DJ/8/2-hSyn1-GCaMp6f-WPRE (titer: 4.5x10E12 vg/ml), ssAAV-5/2-hSyn1-iGluSnFR(A184S)-WPRE (titer: 5.3x10E12 vg/ml) were purchase from the UZH Vector Facility (Zurich, Switzerland). AAV9-hSyn-5HT3.5 (titer: 1x10E13 vg/ml), AAV9-hSyn-ACh3.0 (titer: 1x10E13 vg/ml) were purchased from BrainVTA (Wuhan, China). pGP-AAV5-syn-iGABASnFR2-WPRE (titer: 2.67x10E13 vg/ml) was provided by the GENIE Project at HHMI Janelia Research Campus (10.25378/janelia.19709311.v3). rAAV8-hSyn1-Jaws-GFP (titer: 1.3x10E13 vg/ml) was purchased from Addgene.

### Stereotaxic surgeries for viral injections

Mice were anesthetized with ketamine (150 mg/kg)/xylazine (10 mg/kg) (Cantonal University Hospital, Lausanne, Switzerland). The surgery was performed using the ocular protector Viscotear to prevent eye damage, a heating pad to keep a stable body temperature, and local anesthesia (subcutaneous injection) with a mix of lidocaïne (6 mg/kg) and bupivacaine (2.5 mg/kg). We unilaterally or bilaterally (when required for the experiment, i.e. optogenetics in vivo) injected in the LHb (−1.4 mm AP, 0.45 mm ML, 3.1 mm DV) 150–250 nl of virus using a glass pipette on a stereotactic frame (Kopf, France). All injections were performed at a rate of approximately 100–150 nl/min. The injection pipette was withdrawn from the brain 10 min after the infusion. Animals were allowed to recover for a minimum of two weeks before fiber or GRIN lens implantation.

### Chronic implants

For fiber photometry experiments, a single fiber probe (200 μm, Chi Square Bioimaging) was placed and fixed (C and B Metabond, Parkell) 100 μm above the injection site in the LHb. For optogenetic manipulation a single fiber (200 μm, Thorlabs) was placed at the following coordinates (AP: −1.4 mm, L: ± 0.1 mm, V: −2.2 mm). Surgery was performed under isoflurane anesthesia (induction: 4%, maintenance: 1.8–2%, Univentor). For endoscope experiments, mice were anesthetized (as described above) and implanted with a GRIN lens (6.1 mm length, 0.5 mm diameter; Inscopix, #100-000588). The lens was placed ∼150–200 μm above the injection site using the following coordinates from bregma (−1.40 mm AP, 0.45 mm ML, −2.85 to −2.9 mm DV; lowered at a speed of 1 μm/s). A stainless steel headbar was implanted on the skull. To do so, the skull was scraped clean and covered with a layer of Cyanoacrylate glue (Vetbond, 3 M). The headbar was lowered to touch the skull over lambda, then secured to the skull with a layer of dental adhesive (C and B Metabond, Parkell), followed by dental cement (Jetkit, Lang). For pain management, paracetamol (500 mg/250 ml; 200-300 mg/kg/day) was added to the drinking water after the surgery. Proper viral expression and fiber/GRIN lens placement in brain areas of interest were confirmed post hoc using histology for all experiments.

### Histology and immunohistochemistry

For histology, mice were terminally anesthetized with ketamine and xylazine or pentobarbital and perfused transcardially with paraformaldehyde (PFA) 4% in 0.1 M phosphate-buffered saline (PBS). Brains were collected and left overnight in 4% PFA at 4 °C until slicing. Consecutive coronal slices (60–100 microns) were sectioned using a vibratome (Leica VT1200S). We took images with an epi-fluorescent microscope (Zeiss) to confirm efficient targeting of the LHb, and we discarded mice whenever this was not achieved (fiber optic, GRIN lens misplacement, or not targeted viral expression).

**Fig. 1:**
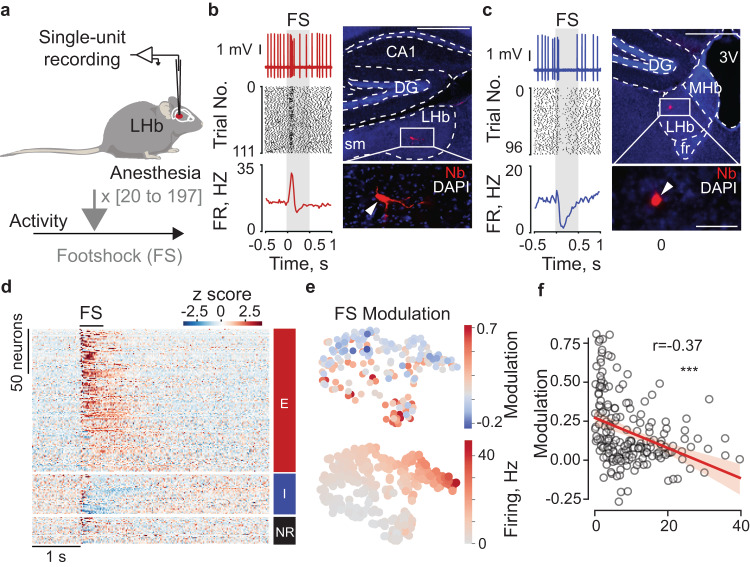
LHb neurons can show excitatory or inhibitory responses to foot shock. **a** Experimental protocol related to the extracellular single-unit electrophysiological recordings in anesthetized mice; for each recorded neuron, mice received between 20 and 120 contralateral foot shocks. **b** Electrophysiological trace, raster plot, average firing response, and picture of an example foot shock-excited neuron labeled in the LHb (scale bar top=500 µm, bottom=100 µm). **c** Same as b but for a foot shock-inhibited neuron. **d** Heatmap of single neuron z-scored average response to FS presentation (n_neurons_=196; n_neurons_ EXC = 134, n_neurons_ INH = 37, n_neurons_ NR = 25). **e** t-SNE plots with superimposed color-coded FS-modulation index (top) and baseline firing rate (bottom). **f** scatter plot with linear correlation of firing rate and modulation index with all cells (n_neurons_ = 196, ^***^p < 0.001).

Juxtacellularly labeled neurons (as in Fig. 1B, C) were visualized according to previously published procedures [23]. Specifically, brains were sliced on a vibratome (VT1200S; Leica) to obtain 50–70 μm thick parasagittal or coronal sections. Brain slices were processed with streptavidin conjugates (streptavidin-546 or -488, Invitrogen). Fluorescence images were acquired by epifluorescence microscopy (Axio imager; Zeiss) and confocal microscopy (LSM 900; Carl Zeiss, Oberkochen, Germany). To calculate a relative arbitrary fluorescence (RAF) of Jaws expression in LHb and neighboring regions, green signal intensity was normalized against a region containing no green fluorescence (background) using the formula: (signal - background/signal + background).

For immunohistochemical analysis, brain sections were permeabilized at room temperature (RT) in 0.5% Triton X-100 (Sigma), followed by 1 h RT blocking in 2% BSA 0.5% Triton X-100 and overnight incubation with primary antibodies (EAAC1, AB1520 Millipore, 1:100; GAD67, MAB#5406 Clone 1G10.2, 1:1000) at 4 °C. Upon washing, sections were incubated for 2 h RT with Alexa-fluorophore-conjugated secondary antibodies (Invitrogen). Confocal microscopy was performed with a Stellaris 5 (Leica) Laser Scanning System and images were processed and analyzed by FIJI/ImageJ Software. Confocal z-stack projections of confocal micrographs for GCaMP6f-GFP signal and for EAAC1 or GAD67 immunofluorescence were thresholded and used to manually outline positive cells. Cell masks produced for GCaMP6f and EAAC1 or GAD67 were subsequently multiplied, to generate the counts of double positive cells.

### Pavlovian conditioning

For Pavlovian conditioning, mice were headfixed in a 3 cm-wide acrylic cylinder. The setting was adapted from eye-blink conditioning methodological preparations as follows. A Basler camera (a2A1920-160umPRO or acA1300-60gc) was equipped with a macro-objective (either Fujinon HF9HA-1S or computar M3Z1228C-MP), and infrared LEDs were used to illuminate mice’s face. For behavioral assessment during two-photon calcium imaging, the camera was equipped with a NIR short-pass filter with a 900 nm cutoff (FES0900, Thorlabs) to filter out excitation light. The airpuff was delivered with pressure from a Picospritzer III (Parker) with a 23G needle directed to the right eye (1 cm distance). A speaker to deliver acoustic tones was placed nearby the mice and was controlled by an MP3 trigger WIG-13720 (Sparkfun). The behavioral apparatus was controlled via Arduino custom code. Behavioral task events were transferred onto Excel with PLX-DAQ interface. Facial videography was recorded with a frame rate of 10 Hz.

Before behavior started, mice were handled (1-2 days) and acquainted to head fixation (1-2 days). Behaviour started with 2 sessions of habituation where mice were presented with 30 CS+ (16 kHz trilling sound, 75 dB, duration: 1 s), 30 CS- (10 kHz pure tone, 75 dB, duration: 1 s) and 5 unpredicted US (airpuff, pressure: 4-5 psi, duration: 0.5 s), delivered in random order with an inter-trial interval between 30 and 45 seconds. During conditioning sessions (2-3 days), mice received a total of 30 CS- trials and 30 CS+ trials where US was followed after a delay of 0.5 s from the end of CS. For two-photon recordings, mice were submitted to extra sessions of habituation and conditioning (1 to 5) to maximize data collection. For in vitro recordings, the paired group (P) was trained as described while the unpaired group (U) received the same number of stimuli with the same characteristics, but CS+ and US were never contingent (30 CS+, 30 CS- and 30 unpredicted US trials). For Jaws-mediated optogenetic silencing, 638 nm light (3 mW) was delivered by a laser (Matchbox Integrated Optics) to the LHb through a fiber cable (M83L1, Thorlabs) connected to a fiber optic implanted in the head of the mouse (CMFLC12L05, Thorlabs). Light was delivered during CS+ trials for 5 s starting from cue presentation.

Behavioral assessment was recorded for a total of 15 s each trial with a sample rate of 10 fps starting 5 s before CS or US delivery. Data were analyzed using a custom-made Python script by thresholding the images to get only the eye pixel values. These values were normalized to 100 percent on a trial-by-trial basis in reference to the average value during the 5 s before cue presentation. From these data, the conditioned response (CR) amplitude was computed by averaging the values between the start of cue delivery (time: 0 s) and the start of airpuff presentation (time: 1.5 s). The discrimination score was obtained by subtracting the average value of the CR for CS+ trials to the one of CS- trials.

### Two-photon microscopy

Two-photon microscopy experiments were carried out to visualize LHb neuronal Calcium dynamics in vivo. Recordings and Pavlovian conditioning started when the field of view (FOV) was clear and stable for at least 3 weeks, typically 1-3 months after GRIN lens implantation. The acquisition was performed with a two-photon Ultima Investigator microscope (Bruker) combined with a Chamaleon-Ultra Tunable Laser (Coherent) and equipped with an Olympus 20× air objective (LCPLN20XIR). The two-photon microscope is equipped with a hybrid scanning core set with galvanometers and fast resonant scanners (up to 30 Hz frame-rate acquisition), multi-alkali PMT and GaAsP-PMT photodetectors with adjustable voltage, gain, and offset features, a single green/red NDD filter cube.

Images were acquired at 30 Hz at a resolution of 512 × 512 pixels and we performed an online averaging of 6 times to get an actual frame rate of 5 Hz. The average power of the beam measured at the front of the objective was between 80 and 125 mW. At each trial, recordings started 5 s before cue delivery, and a 15s-long video was collected. Images were collected using a computer equipped with Prairie view (Bruker). Prior experiment started, 1 up to 3 FOV per mouse was chosen to maximize the number of neurons recorded each session by adjusting the imaging plane (z-axis), and each FOV was spaced at least 60 μm from one another to prevent visualization of the same cells across multiple FOVs. For imaging sessions with multiple fields of view, the conditioning session was split, allowing an equal number of trials per FOV. Sessions in which mice did not display any behavioral learning phenotype, were excluded from the analysis.

After data collection, videos were motion-corrected with a planar hidden Markov model (SIMA v1.3.2), and regions of interest (ROIs) were manually drawn around each cell using the standard deviation projection of the motion-corrected video using ImageJ.19. Neurons that were not tracked for at least one habituation and one conditioning session were not kept in the analysis. Next, fluorescence time series data were extracted with ImageJ BAR plug-in and analyzed using custom Python data analysis pipelines. % ΔF/F0 was calculated on a trial-by-trial basis as: ((F − F0)/F0)*100, where F0 is the average of the three seconds preceding the start of the CS presentation. To classify neurons based on their responses to CS and US (inhibited, not-responding or excited), we compared the area under the curve (AUC) during 1.5 s baseline versus the AUC during the 1.5 s anticipatory period (CS and delay) or US presentation (Wilcoxon Rank Sum Test, using a significance threshold of *p* ≤ 0.05). χ2 test was then used to compare the distribution of responses in CS+ and CS- trials, and across sessions.

We performed decoding analyses to determine if neuronal activity could be used to predict the general mouse eyeblink during cue presentation on any given trial. To do so, we used a binary decoder for each neuron where its activity (computed as AUC) during cue presentation (i.e. from 0 to 1.5 s from cue starting) was used to predict whether the eye blinking (as CR) was lower than the 60th percentile or higher than the 40th percentile of that particular session.

To complete the decoding analysis, we used the Python module Scikitlearn with GridSearchCV and a support vector classification (SVC) estimator [24]. This included a radial basis function kernel. Quantification of performance was done using 100 folds validation for each neuron, the average accuracy score across these parameters was used as the metric of accuracy. In order to determine whether the neuron’s accuracy scores across all repetitions were significantly different from that expected by chance, we performed a single shuffle per neuron by randomizing the cue identity on every trial and tested using an unpaired *t*-test.

### Fiber photometry recordings

Fiber photometry recordings were performed using the ChiSquare X2-200 system (ChiSquare Biomaging, Brookline, MA). Briefly, blue light from a 473 nm picosecond-pulsed laser (at 50 MHz; pulse width ∼80 ps FWHM) was delivered via a single mode fiber. Fluorescence emission was collected by a multimode fiber with a sampling frequency of 100 Hz. The single mode and multimode fibers were arranged side by side in a ferrule that is connected to a detachable multimode fiber implant. The emitted photons were bandpass-filtered (FF01-550/88, Semrock) to a single photon detector. Photons were recorded by the time-correlated single photon counting (TCSPC) module (SPC-130EM, Becker and Hickl, GmbH, Berlin, Germany) in the ChiSquare X2-200 system. Photometric recordings were analyzed using a custom made python code where the signal was smoothed (running average: 10) and % ΔF/F0 was calculated on a trial-by-trial basis as: ((F − F0)/F0)*100, where F0 is the average of the three seconds preceding the start of the CS (or US presentation for unpredicted US trials). To determine whether we were able to collect valid signal, for each mouse, we analyzed the averaged response during the habituation session to CS and US presentation. We kept only the mice where we could detect positive or negative photometric responses when the mean number of photons/bin in at least 3 epochs (50 ms per epoch) was higher than the baseline average plus two times the Standard Deviation (SD) or lower than baseline minus two times the SD.

### In vivo electrophysiological recordings

Juxtacellular recordings of single LHb neurons (dataset in Fig. 1) were performed as previously described [14, 25, 26]. A subset of recordings (n_neurons_=149) was obtained in animals anesthetized with a mix of ketamine/xylazine according to previously published procedures [26]. Briefly, craniotomies were performed at stereotaxic coordinates (1.5 mm posterior, 0.5 mm lateral to bregma) above the LHb. Glass electrodes (impedance: 7-9 MOhm) were filled with a tracer-containing solution (1.5–2% Neurobiotin, SP-1120, Vector Laboratories). Juxtacellular voltage signals were acquired (ELC-03XS, NPI Electronic, Tamm, Germany) and digitized at 25 kHz (POWER1401-3 and Spike2 v. 8.02e, CED, Cambridge, UK). Spontaneous spiking was monitored for at least 5 min, followed by foot shock stimulation (−1 mA, 0.5 s, every 5 s, number of trials=20–197 trials) to the contralateral hind limb (A365R Stimulus Isolator, WPI). Juxtacellular labeling was performed using standard procedures. Recordings that were histologically confirmed to be outside of the LHb and recordings containing more than one unit or indications of cell damage were excluded from the analysis. Spikes from juxtacellular signals were isolated through threshold-based peak detection and visual inspection assisted by PCA, essentially as previously described [27]. After recording, animals were euthanized with an overdose of pentobarbital, transcardially perfused and their brains processed for histological analysis as previously described [23] (see also ‘histology and immunohistochemsitry’ above).

Part of the juxtacellular dataset in Fig. 1 was previously obtained (n_neurons_=121) from part of the data included in Congiu et al. 2019 and Lecca et al. 2017. Experimental procedures were carried out as previously described [14, 25]. Briefly, mice were anesthetized using isoflurane (Induction: 4%; maintenance: 1–1.5%) and placed in the stereotaxic apparatus (Kopf, Germany). Their body temperature was maintained at 36 ± 1 °C using a feedback-controlled heating pad (CMA 450 Temperature controller, Phymep). The scalp was retracted, and one burr hole was drilled above the LHb (AP: −1.3 to −1.6 mm, L: 0.35–0.5 mm, V: −2.3 to −3.2 mm) for the placement of a recording electrode. Single-unit activity was recorded extracellularly using glass micropipettes filled with 2% Chicago sky blue dissolved in 0.5 M sodium acetate (impedance 5–15 MΩ). The signal was filtered (band-pass 500–5000 Hz), pre-amplified (DAM80, WPI, Germany), amplified (Neurolog System, Digitimer, UK), and displayed on a digital storage oscilloscope (OX 530, Metrix, USA). Experiments were sampled on- and offline by a computer connected to CED Power 1401 laboratory interface (Cambridge Electronic Design, Cambridge, UK) running the Spike2 software (Cambridge Electronic Design).

Single units were isolated, and the spontaneous activity was recorded for a minimum of 3 min before assessing their response to foot shock delivery (duration: 0.5 s, intensity: 1 mA, ITI: 5 s). At the end of each experiment, mice were euthanized (overdose of isoflurane prior to killing) and the electrode placement was determined with an iontophoretic deposit of pontamine sky blue dye (1 mA, continuous current for 5 min). Brains were then rapidly removed and fixed in 4% paraformaldehyde solution. The position of the electrodes was identified with a microscope in coronal sections (60 μm). Only recordings in the correct area were considered for analysis.

### In vitro electrophysiology

Mice were anesthetized (ketamine/xylazine; 150 mg/100 mg kg−1), sacrificed, and their brains were transferred in ice-cold carbogenated (95% O2/5% CO2) solution, containing (in mM) choline chloride 110; glucose 25; NaHCO3 25; MgCl27; ascorbic acid 11.6; sodium pyruvate 3.1; KCl 2.5; NaH2PO4 1.25; CaCl20.5. Coronal brain slices (250 μm thickness) were prepared and transferred for 5 min to a warmed solution (34 °C) of identical composition, before transfer at room temperature in a carbogenated artificial cerebrospinal fluid (ACSF) containing (in mM) NaCl 124; NaHCO3 26.2; glucose 11; KCl 2.5; CaCl2 2.5; MgCl2 1.3; NaH2PO4 1. During recordings, slices were continuously superfused with ACSF at a flow rate of 2.5 mL min^−1^ at 32 °C. Neurons were patch-clamped using borosilicate glass pipettes (2.7–4 MΩ; Phymep, France) under an Olympus-BX51 microscope (Olympus, France). The signal was amplified, filtered at 5 kHz and digitized at 10 kHz (Multiclamp 200B; Molecular Devices, USA). Data were acquired using Igor Pro with NIDAQ tools (Wavemetrics, USA). Access resistance was continuously monitored with a − 4 mV step delivered at 0.1 Hz. Extracellular stimulation from AMPI ISO-Flex stimulator was delivered through glass electrodes placed in the LHb. All recordings were made in voltage-clamp configuration. For AMPA/GABA evoked excitatory currents were recorded at −60mV, and evoked inhibitory currents were recorded at +5 mV. AMPA/NMDA ratios were obtained recording compound AMPA + NMDA EPSCs at +40 mV, and subtraction of the APV-insensitive component (APV, 100 μM). The paired-pulse ratio was obtained by recording the neurons at −60 mV and by delivering two pulses at 50 msec interpulse intervals. Spontaneous excitatory currents were pharmacologically isolated by bath application of picrotoxin (PTX, GABAAR antagonist; 100 μM). The internal solution contained (in mM) CsMeSO3 120, CsCl 10, HEPES 10, EGTA 10, creatine phosphate 5; Na2ATP 4; Na3GTP 0.4, QX-314 5. All drugs were purchased from HelloBio.

### Real time place aversion/preference test

C57BL/6 J wild-type male mice 8–10 weeks old were tested in a custom-made behavioral arena with two compartments with different visual cues and wall texture for 15 min. The time spent on each compartment was recorded via a digital camera interfaced with Ethovision software (Noldus). Paired-side for the CS+ auditory tone was randomly assigned and balanced among experimental subjects and groups. Mice were habituated for one session to the tone to avoid novelty-induced effects. At the beginning of the test session, the mouse was placed on the non-paired side of the chamber. Every time the mouse crossed to the paired side CS+ tone was played (duration 1 s; ITI 5 s) via a hardware timed-signals controlled by an I/O box (Noldus). The sound was interrupted at any instance at which the mouse left the paired side.

### Quantification and statistical analysis

All statistical analyses were conducted using Prism (v.9, GraphPad) or the Python scikit-learn library. Statistical tests used in this study include paired and unpaired *t*-test, Wilcoxon matched-pairs tests, Chi-Square test, one-way and two-way analysis of variance (ANOVA). When parametric tests were used, data normality was confirmed using the Shapiro–Walk normality test. *P* values were corrected for multiple comparisons when necessary. In the box plots, the center lines indicate the median, and the box limits indicate the upper and lower quantiles (95%); box plots include single observations. The significance threshold was held at α = 0.05 and tests were two-sided. Sample sizes were not predetermined using statistical methods but based on experimental settings within the laboratory. Experiments were randomized whenever possible. Experimenters were not blind to the experimental group apart from the in vitro electrophysiological recordings. Experiments were replicated at least two times within the laboratory. t-SNE embeddings were generated using 15 electrophysiological features: the first four principal components of interspike interval distributions (ISIs; bin size: 10 ms; computed for a range of 1 s), the first four principal components of autocorrelograms (ACGs; bin size: 1 ms; computed for a range of 500 ms and a narrower range of 100 ms), spontaneous firing rate, coefficient of variation (CV; ISI standard deviation/mean ISI, as in Softky and Koch 1993), and burst index (fraction of ISIs smaller than 25 ms). To classify neurons as footshock excited, inhibited, and non-modulated, we compared the baseline firing rate (FR_baseline_; 1 s prior to stimulus onset) with the stimulus firing rate (FR_stimulus_; 1 s after stimulus onset). Out of the 220 neurons with footshock stimulation, only neurons with a >20 footshock stimulating trials (n_neurons_ = 196) were included in this analysis, in order to have adequate statistical power. Neurons were classified as FS-excited or FS-inhibited by comparing FR_stimulus_ and FR_baseline_ (Wilcoxon rank sum test, alpha = 0.5); neurons with FR_stimulus_ > FR_baseline_ were assigned to the FS-excited group and neurons with FR_stimulus_ < FR_baseline_ were instead assigned to the FS-inhibited group. To quantify the strength of foot shock modulation, we computed a foot shock modulation index (FS-modulation) defined as: (FR_stimulus_ – FR_baseline_) / (FR_stimulus_ + FR_baseline_).

## Results

### Punishment drives distinct LHb neuronal populations with excitation or inhibition

Punishments generate excitation of LHb neurons as measured by recordings of neuronal activity in anesthetized mice and analysis of bulk calcium (Ca^2+^) signals through fiber photometry [7, 10, 14]. This led to a general framework whereby LHb encodes aversion through its excitation. However, recent work indicates that the response properties of individual LHb neurons to aversive stimuli might be more heterogeneous [25, 28, 29]. To systematically explore this possibility, we performed juxtacellular recordings from single LHb neurons in anesthetized mice, while delivering foot shock stimuli (Fig. 1a). In line with previous observations [25, 30], we found that foot shock stimuli led to a rapid increase in spiking activity in a majority of LHb neurons (Fig. 1b). Notably, these excitatory responses were heterogenous in their kinetics likely due to diverse ionic conductances or synaptic input organization onto LHb cells (Supplementary Fig. S1a–f). Aside from the foot shock-excited population, a distinct subset of LHb cells was instead significantly inhibited by the stimuli (Fig.1c, d). To explore whether foot shock responses relate to in-vivo firing patterns of LHb neurons, we used Stochastic Neighbor Embedding [31] and visualized the neurons in two-dimensional space (Fig. 1e; see Methods). Mapping of foot shock responses onto the tSNE embedding, which was created based on spontaneous spiking features alone (see Methods), revealed a gradual organization of foot shock responses that correlated with spontaneous firing rates (Fig. 1e). Indeed, foot shock modulation and spontaneous firing rates were negatively correlated (Fig. 1f; [25]). Thus, these data indicate that in the LHb, punishments generate rapid and opposing responses following a non-random organization where foot shock excitation is more common in low-firing neurons while inhibition in high-firing cells. Altogether, this diversity of responses expands the current predominant framework in which the LHb encodes aversion solely through neuronal excitation.

### Disrupting LHb function affects cue discrimination during punishment associative learning

**Fig. 2:**
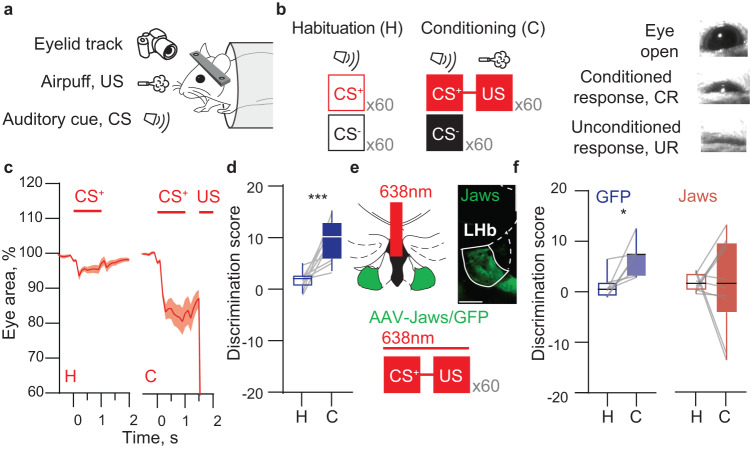
LHb integrity is necessary for cue discrimination during Pavlovian conditioning. **a** Experimental protocol of the behavioral setup for the Pavlovian conditioning task in awake head-restrained mice. **b** Left, schematic representing the structure of conditioning; right, example pictures of the behavioral responses during the task **c** Time course of the average eye area changes across CS+ trials during habituation (H) and conditioning (C) (n_mice_=10). **d** Box-plot of the discrimination score during habituation and conditioning (n_mice_=10; paired *t* test, t_9_ = 4.92, ^***^p < 0.001). **e** Top, schematic of optogenetic inhibition and representative coronal section of Jaws expression in the LHb and fiber track (scale bar: 200 µm); bottom, schematic of the experimental protocol for optogenetic LHb inhibition. **f** Box-plots of the discrimination score for GFP mice (n_mice_=7; paired *t* test, t_6_ = 3.20, ^*^p = 0.019) and Jaws mice (n_mice_=10; paired *t* test, t_9_ = 0.059, *p* = 0.954).

To understand whether the functionally diverse signatures of LHb activity in relation to punishments hold true in awake and behaving mice, we modified a classical conditioning task designed for investigating value coding in dopamine neurons with the objective of studying neuronal dynamics at a single-cell resolution [1, 4]. Head-fixed mice were trained to associate one conditioned auditory stimulus (CS+), but not another (CS−), with an airpuff directed to the eye (Fig. 2a, b). Following two sessions of habituation to the auditory cues and two conditioning sessions where only the CS+ was contingent to airpuff presentation, mice displayed anticipatory eyeblink to the CS+ only, indicative of CS–US association (Fig. 2c; Supplementary Fig. S2a–d). To quantify congruous learning, we tracked and analyzed the eye area during CS to compute the discrimination score (CS– average - CS+ average), a measure significantly higher after conditioning compared to habituation (Fig. 2d; Supplementary Fig. S2e, f). We next aimed to probe the necessity of LHb for cue discrimination during associative learning by optically reducing its activity. We transduced LHb neurons with a light-driven chloride pump (orange-red spectrum of activation) via the infusion of rAAV8-hSyn1-Jaws-EGFP (Fig. 2e). Four weeks later, we assessed basal LHb neuronal activity using single-unit recordings in anesthetized mice [14]. Light at 638 nm produced a time-locked reduction in the basal firing rate of LHb neurons (Supplementary Fig. S3a–d). Thus, Jaws efficiently reduces LHb neuronal activity. Next, we chronically implanted Jaws-expressing mice with a single fiber optic above the LHb (Fig. 2e; Supplementary Fig. S3e, f). Shining light at 638 nm to inhibit LHb function throughout CS+ and US presentation diminished cue discrimination during conditioning (Fig. 2f; Supplementary Fig. S3g). We acknowledge minimal Jaws spread in the paraventricular nucleus of the thalamus (PVT), a structure as well relevant for cue-punishment association [32]. Although we cannot completely rule out PVT contribution in our task, the presented experimental evidence, the depth of red-light penetration, and the location of fiber probes support the LHb as a player in establishing behavioral cue discrimination emerging during associative learning.

### LHb neuronal populations develop opposing responses to punishment-predictive cues across learning

**Fig. 3:**
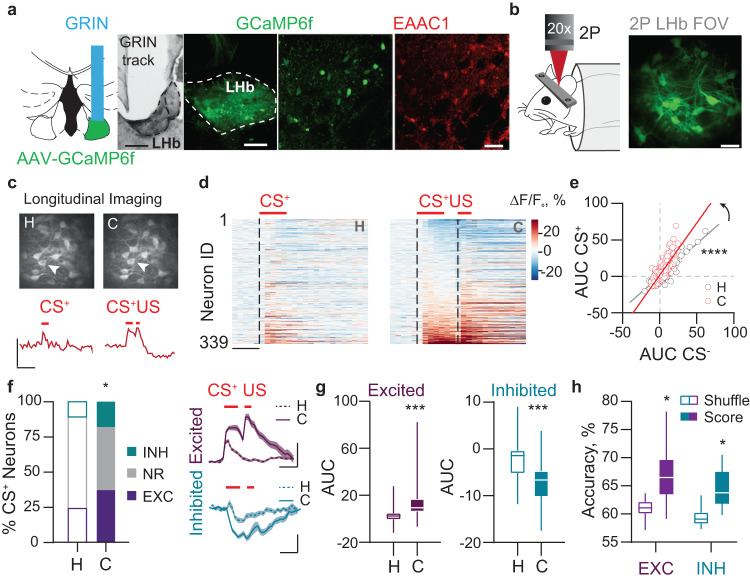
Single neuron inhibitory and excitatory responses adapt during associative learning. **a** Schematic of the experimental preparation for the LHb two-photon calcium imaging, example coronal histological section of GRIN lens positioning above LHb (scale bar: 200 µm), GCaMP6f expression on LHb neurons (scale bar: 100 µm) and staining of the EAAC1 glutamatergic marker (scale bar: 30 µm). **b** Schematic representation of the experimental setting for two-photon calcium imaging recordings in head-restrained awake mice (left) and standard deviation projection of an example field of view (FOV) (right). **c** Example of longitudinal tracking of the same FOV across habituation and conditioning sessions (top) and average response of the indicated neuron (arrowhead; bottom; scale bar: 10 ΔF/F0 %, 3 s). **d** Heatmaps of the average ΔF/F0 response of single neurons during habituation (left) and conditioning (right) (scale bar: 1 s). **e** Scatter plot of the area under the curve (AUC) during CS+ and CS- for habituation (white dots) and conditioning (red dots) (F_(1,674)_ = 86.76, ^*^p < 0.0001, n_mice_ = 12, n_neurons_ = 339). **f** Left, contingency table of the proportion of neurons statistically inhibited, not responding or excited during CS+ presentation (Χ2 = 8.10, ^*^p = 0.018); right, time-course of the grand-average responses to CS+ trials during habituation and conditioning trials for the excited (top, n_neurons_ = 118) and inhibited neurons (bottom, n_neurons_ = 45). **g** Box-plot of the population average AUC response during CS+ presentation for the excited (left; paired t-test, t_117_ = 9.08, ^***^p < 0.0001) and inhibited neurons (right; paired *t*-test, t_44_ = 6.70, ^** *^p < 0.0001). **h** Box-plot of the accuracy score for the excited and inhibited neurons (n_cells_: excited=47; inhibited=10; Two Way ANOVA RM and Sidak’s multiple comparison test, shuffle vs score F_(1,55)_ = 93.84, ^*^p < 0.0001).

To track LHb neuronal activity in behaving mice throughout Pavlovian conditioning, we injected a viral construct encoding the Ca^2+^ indicator GCaMP6f (rAAVdj-GCaMP6f) into the LHb [7]. Next, we implanted a microendoscopic lens allowing chronic optical access to the LHb through a two-photon microscope equipped with a 20X air objective (Fig. 3a–c). Post hoc analysis reflected that the majority of the LHb neuronal population expressing GCaMP6f was glutamatergic and not GABAergic (i.e., EAAC1+ and GAD67–; Fig. 3a; Supplementary Fig. S4a–c; [7]). We recorded from GCaMP6f-expressing LHb neurons across multiple fields of view (FOVs) within each mouse (*n* = 12 mice, 21 FOVs, 339 neurons; Fig. 3d). We computed cue-driven neuronal responses and found that, while a population of LHb neurons displayed a substantial increase in their fluorescence, a different neuronal ensemble exhibited a reduction in fluorescence after the presentation of the punishment-predictive cue during conditioning, compared to the habituation period (Fig. 3d). Notably, CS + -driven neuronal responses before conditioning were likely due to the saliency of the stimulus and not an intrinsic aversive component of the auditory cue. Indeed, mice did not show avoidance in a real-time place paradigm test (Supplementary Fig. S5a). Notably, CS+ responsive neurons were also tuned in the same direction by the air puff (Supplementary Fig. S5b, c). Tracking and quantification of fluorescence signals during the presentation of CS+ and CS– were indicative of plasticity after conditioning (Fig. 3e). Indeed, we found that throughout habituation to conditioning, 121/339 neurons displayed an increase in Ca^2+^-mediated fluorescence (likely corresponding to higher excitation) time-locked to the CS+, whereas 47/339 showed a significant reduction in fluorescence to the CS+ across learning (likely corresponding to larger inhibition; Fig. 3f, g; *P* ≤ 0.05, CS-evoked signals versus baseline, rank-sum test). Thus, distinct populations of LHb neurons developed an enhancement of existing excitatory or inhibitory responses as well as new excitatory or inhibitory responses to a punishment-predictive cue across learning.

Different functional responses in the LHb may anchor on territorial distribution, which embeds LHb neurons within independent circuit connectivity loops [12]. To examine the anatomical localization of excited and inhibited neurons, we matched the fields of view of GCaMP6f-expressing neurons with the respective Gradient Index (GRIN) lens edges and the anatomical borders of LHb (See methods). CS+-activated neurons undergoing potentiation after learning largely overlayed the lateral territory of the LHb. In contrast, CS+-inhibited neurons after conditioning were rather medially-located (Supplementary Fig. S5d, e). Notably, when preparing acute brain slices and patching neurons located in the lateral aspect of the LHb after CS-airpuff association, we observed increased AMPA/GABA and AMPA/NMDA ratios along with larger amplitudes of spontaneous excitatory currents (sEPSCs) compared to control mice (CS and airpuff non-contingent; Supplementary Fig. S6a–e). In contrast, the paired-pulse ratio of evoked AMPA current and the frequency of sEPSCs remained unaltered (Supplementary Fig. S6d, e). Altogether, distinct LHb neurons undergo a CS+-driven increased excitation or inhibition, are anatomically segregated, and develop postsynaptic adaptations across punishment associative learning.

We then predicted that cue responses developing through learning may represent information related to eye blinking and, therefore, cue discrimination (CS+ versus CS–). Using the CS-driven responses of each LHb neuron within each trial, we trained a support vector machine (SVM) decoder to predict, on a trial-by-trial basis, whether or not mice displayed a conditioned response. We found that activity dynamics of subpopulations of excited and inhibited LHb neurons predicted whether animals’ eye-blinked during the cue as compared to the shuffled data from the same neuron (Fig. 3h). In line with the observation that optically perturbing LHb function disrupts cue discrimination, these findings suggest that cue responses in LHb neurons represent information related to the discrimination of independent cues, and therefore proper cue-punishment association.

### Acetylcholine, but not glutamate, GABA and serotonin, release in LHb mirrors neuronal dynamics after conditioning

**Fig. 4:**
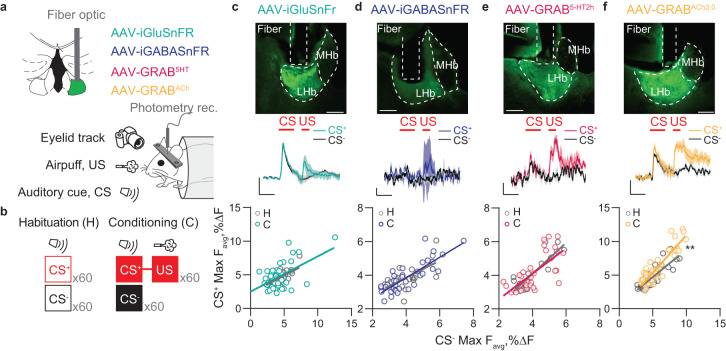
Presynaptic ACh release in the LHb, but not glutamate, GABA, or serotonin, reflects cue discrimination. **a** Schematic of experimental setup for the photometric recordings of neurotransmitter biosensors dynamics in the LHb. **b** Schematic of the experimental setting in head-restrained mice. **c–f** Top, histological example of the expression of the neurotransmitter sensor and fiber placement in the LHb; middle, grand-average time-course of the ΔF/F0 response for CS+ and CS-; each trace is normalized by the maximum amplitude of the CS- response (n_trials_: CS+=60, CS−=60, legends =1%, 1 s); bottom, scatter plot of the average peak ΔF/F0 response for CS+ and CS- on a 5 trial bin for habituation and conditioning trials (C, iGluSnFR, n_mice_ = 5, F_(1,86)_ = 0.08, *p* = 0.77; D, iGABASnFR2, n_mice_ = 3, F_(1,50)_ = 0.39, *p* = 0.53; E, GRAB5-HT2h, n_mice_ = 3, F_(1,50)_ = 0.59, *p* = 0.45; F, GRABACh3.0, n_mice_ = 6, F_(1,116)_ = 8.13, ^*^p = 0.0052).

Synaptic adaptations within the LHb after learning rely on postsynaptic mechanisms, including AMPA or GABA receptor trafficking/efficiency rather than changes in glutamate or GABA release [7, 33]. On the other hand, neuromodulators including serotonin and acetylcholine (ACh), are capable of modulating synaptic function and contributing to motivated behaviours [34–36]. Thus, it is plausible that during cue-punishment Pavlovian conditioning, while glutamate and GABA release remain stable, neuromodulation would instead adapt and match the postsynaptic responses, potentially representing a gating mechanism for plasticity and learning. To test this possibility, we leveraged recent advances in the readout of fluorescence intensity-based genetically encoded sensors with fiber photometry in head-fixed mice during the Pavlovian discrimination task [30]. The genetically encoded sensor iGluSnFR enables glutamate release detection, the iGABASnFR2 detects GABA release instead, while GRAB^5-HT2h^ and GRAB^ACh3.0^ permit measurement of serotonin and ACh, respectively [37–41]. We expressed each of the sensors in an independent group of mice through viral injections in the LHb (Fig. 4a). Presentation of an unpredicted airpuff or a neutral auditory cue produced rapid glutamate, GABA, serotonin, and ACh transients (Supplementary Fig. S7a–d). During conditioning, iGluSnFR, iGABASnFR2, and GRAB^5-HT2h^-mediated fluorescence transients remained comparable between CS+ and CS– (Fig. 4b–e and Supplementary Fig. 7a–c). Instead, similarly to the postsynaptic GCaMP6f responses, ACh transients significantly increased after learning in response to CS+ compared to CS– (Fig. 4f and Supplementary Fig. 7d). This indicates that postsynaptic adaptations of CS+-driven excitatory and inhibitory responses in the LHb during Pavlovian conditioning occur along with a presynaptic increase of cholinergic signaling, but not glutamate, GABA and serotonin transmission.

## Discussion

This study reveals unprecedented and remarkable diversity in neuronal responses to punishments and their predictive cues in the LHb, unraveling complex opposing signals which may contribute to negative affect-related behaviours in health and disease.

We found that LHb neurons, generally thought to be excited by aversive events, physically respond with excitatory or inhibitory signals to punishment as measured by in vivo recordings from identified LHb neurons. To extend the behavioural relevance of these findings, we show that an appropriate function of LHb is required for proper cue discrimination in a Pavlovian task where mice learn to associate a specific cue to an upcoming punishment. LHb neuronal ensembles longitudinally recorded during learning expressed opposing cue-driven responses and adaptations with enhanced excitation and inhibition. Our data reveal that changes in excitatory and inhibitory responses in distinct LHb neuronal populations guide cue-punishment associations and consequent anticipatory behavior. These postsynaptic adaptations mirror the dynamics of specific presynaptic signaling onto LHb neurons whereby the steady glutamate and GABA release contrasts with plastic ACh signaling. These data identify novel neuronal mechanisms underlying negative valence encoding in the LHb. More broadly, our findings suggest that LHb responses to aversive stimuli, often considered as homogenously excitatory, are instead diverse and plastic and that, along with neuromodulatory signaling in LHb, adapt throughout learning processes. Altogether, this reflects specialized and complex LHb organization and functions which likely coordinate specific behavioral outcomes (i.e. anticipatory behaviours).

The LHb responds to aversive external stimuli with a phasic increase in neuronal activity in both awake and anesthetized animals [5, 10, 14], a functional feature independent of the nature of the negative stimulus. Accordingly, foot shock, air puff, and radiant heat promote excitation of the LHb [7, 42, 43]. Thus, excitation in the LHb is defined to encode negative valence, and, if persistent, to underlie negative affect in psychiatric states [12]. In the present work, we systematically characterized neuronal responses in the LHb by using in-vivo juxtacellular recordings during foot shock stimulation in anesthetized animals. Our dataset of ~200 neurons extends previous observations [14, 25] and shows that neuronal responses to foot shock are diverse, and represented by both excitation and inhibition [25]. Notably, a similar diversity of responses was also observed in awake animals [30]. After recording more than 300 LHb cells during a Pavlovian task under a two-photon microscope, the analysis recapitulates the functional diversity and unravels how cue-driven excitation and inhibition relate to punishment-mediated transients at the single-cell level. Notably, single-cell responses (both excitatory and inhibitory) to cues were present already during the habituation session, suggesting that LHb neurons may encode the salience of a stimulus and not solely its valence. This is in line with work performed both in rodents and non-human primates [30, 44]. In the latter, phasic salience-related signals are described in the habenula-dopamine pathway – a process that motivates anticipation [44], thus overall supporting the data presented within this work.

The cue-driven excitation and inhibition emerging across the LHb both during habitation and conditioning raise the question of which determinants underlie this diversity. A plausible scenario is that excited and inhibited cells are part of distinct neuronal networks. Reconstruction of the anatomical position of each neuron recorded under the two-photon microscope unravels that inhibited cells occupy the medial territory of the LHb [25], conversely to the excited population located rather laterally. Subcortical innervation onto LHb follows a topographical map with synaptic inputs from the basal ganglia impinging specifically onto the lateral division and the bed nucleus of the stria terminalis rather limited to the medial aspect [15, 16, 34]. Similarly, medially-located neurons synapse downstream to midbrain dopamine neurons and raphe serotonin neurons, while lateral LHb cells send their axons to midbrain GABA neuronal populations [14, 45]. Thus, connectivity-based differences may underly functional signatures for learning in LHb.

In addition, or alternatively, the functional diversity may anchor on transcriptomics differences whereby independently of the neuronal circuit integration, excited and inhibited neurons may differ based on their genetic nature [19, 21, 46]. It remains yet challenging to differentially label neuronal populations expressing distinct plastic responses and discretely study their anatomical organization, function, and molecular identity. The further amelioration of Ca^2+^-, neuronal-activity-, immediate early gene-driven labeling is required to fill this gap, at least in relation to the excited cells [47–49].

The tracking of the neuronal dynamics across the conditioning and the subsequent analysis unravels that a proportion of both inhibited and excited neurons represent cue discrimination and, thereby, CS+-driven anticipatory behaviours. This finding matches the observed loss of cue discrimination after LHb optical inhibition throughout learning. Notably, manipulating LHb function affects cue discrimination in rats undergoing a cocaine-seeking paradigm [50, 51] or in mice during an odor-outcome association task [52]. Altogether, this converges to clarify the contribution of the LHb in enabling discrimination among valued external cues. This offers a framework in which LHb aberrant activity may produce cue generalization – a behavioural feature of disorders including post-traumatic stress disorders, for instance. Behavioural cue discrimination after conditioning occurs along with i. a shift from a silent to a responsive state of a proportion of neurons that become either inhibited or excited and ii. the CS+-mediated increase of both inhibition and excitation. Thus, the CS+-driven plasticity of excited and inhibited cells after conditioning occurs following the direction of the initial CS+-mediated response during the habituation period. This process is different from other subcortical structures, including the amygdala, where adaptations in cue-driven responses after learning remain independent from the initial CS-mediated responses at baseline [22].

The plasticity occurring in the LHb after cue-punishment association may have a synaptic foundation, as suggested by the strengthening of neurotransmission onto cells located in the lateral aspects of LHb. These data are in accordance with a scenario in which learning to anticipate an aversive event requires synaptic potentiation of AMPA-mediated transmission in the LHb [6, 7]. While plasticity at excitatory synapses emerges on putative excited neurons, whether increased inhibition during learning takes place along with adaptations of synaptic GABAa neurotransmission remains to be established. This is plausible, however, as potentiation of synaptic inhibition in the LHb contributes to associative processing [33]. Altogether these data support the notion that postsynaptic adaptations mediate the establishment of cue discrimination and cue-punishment association. This is further corroborated by the observation that presynaptic glutamate and GABA signaling remain comparable between CS+ and CS–. This indicates that presynaptic fast neurotransmission does not change to support cue discrimination, which recapitulates previous findings obtained from recordings in acute brain slices [7, 33]. Our data lack, however, synaptic input specificity, thus presynaptic plasticity events that are synapse-specific cannot be ruled out. For instance, vesicular glutamate transporter-2 (Vglut2)-expressing hypothalamic neurons that project to the LHb undergo adaptations of cell dynamics during fear learning [6]. The differences between behavioural tasks (Pavlovian punishment task in this work versus fear conditioning) or the lack of input-specific assessment might be among the reasons explaining the absence of changes in glutamate release described here.

Synaptic plasticity requires, in many instances, gating processes often mediated by neuromodulatory signaling [53, 54]. Indeed, neuromodulation can prime synapses for the induction and expression of long-term synaptic adaptations [54, 55], whereby cholinergic, serotoninergic, and dopaminergic signaling can control synaptic gain [56–58]. By using genetically encoded sensors for neurotransmitters in the LHb of behaving mice we detected phasic release of ACh and serotonin. While serotonin release represents a relevant modulatory pathway in the LHb [12, 34, 35], and its release occurs along cue and punishment presentation, this does not contribute to cue discrimination, as CS+ and CS– mediated serotonin transients remained comparable during conditioning. Experimental evidence reporting ACh release in LHb during animal behaviour was so far absent. We report the unprecedented observation that ACh transients were detectable during cues and punishment presentation, raising a scenario whereby such signaling emerges during the encoding of salient events independently of their value. In addition, ACh signaling adapts throughout cue-punishment association, mirroring the plastic adaptations observed with GCaMP6f recordings. Thus, ACh release may gate synaptic plasticity in the LHb to support conditioning. While this remains the first attempt to describe ACh presynaptic dynamics, information on the postsynaptic machinery required to convey cholinergic signaling in the LHb already exists. LHb neurons become depolarized or hyperpolarized by activation of muscarinic cholinergic receptors. In addition, muscarinic receptor inhibits synaptic GABA and glutamate transmission [36]. Furthermore, blockade of LHb muscarinic signaling impaired operant cocaine behaviours in a Go/No-Go task [51]. Altogether, this suggests that functional cholinergic signaling through muscarinic receptors in the LHb regulate cocaine-mediated behavioural outcomes. It remains to be established which circuit connectivity underlies ACh release in the LHb, yet our findings highlight the importance of studying cholinergic modulation of synaptic efficacy and its role in behaviours associated with aversive states.

In summary, our findings reveal functional diversity, plasticity events, and neuromodulatory mechanisms in the LHb that contribute to associative learning, cue-punishment association, and cue discrimination – further supporting the relevance of LHb for valence encoding. We postulate that anticipation of upcoming punishments relies on excited and inhibited LHb neuronal populations which play a synergistic role in coordinating learning. This provides insights into how the brain transforms neutral stimuli into punishment-predictive ones.

### Supplementary information

Suppl Mat

## Data Availability

All data are available in the main text or the supplementary materials. Raw material and code for analysis are available upon request to authors and online through the following Zenodo repository 10.5281/zenodo.7784977.
