# Supplementary material for: Plasticity of neuronal dynamics in the lateral habenula for cue-punishment associative learning
Source: Mol Psychiatry. 2023 Jul 6;28(12):5118–27. doi: 10.1038/s41380-023-02155-3 (PMC11041652; doi:10.1038/s41380-023-02155-3)
Supplement: Supplementary file 1 — Suppl Mat [file 41380_2023_2155_MOESM1_ESM.pdf]

Supplementary material

Plasticity of neuronal dynamics in the lateral habenula for cue-punishment associative learning.

Supplementary Figures S1-S7

Supplementary Fig. S1. Kinetics heterogeneity of FS-excitatory responses in LHb neurons.

(a) Z-scored peristimulus time histogram for all FS-excited neurons in the LHb (same as in Fig. 1d) divided in four equally-sized groups. Same n and conventions as in Fig. 1d. **(b)** Mean peristimulus time histograms for the four groups indicated in (b). **(c-f)** Quantification of FS-response properties for the four groups indicated in (a, b). ‘Area under the curve” calculated as the summed z-scored firing rate for every trial within the 0-3 s window following FS onset; ‘Center of Mass’ calculated as the area under the curve weighted by the time vector and divided by the number of bins; ‘Peak Amplitude’ represents the normalized maximal response amplitude within the 0-3 s window following FS onset; ‘Time-to-Peak’ represents the time from stimulus onset to the response maximum. Same color code as in (b).

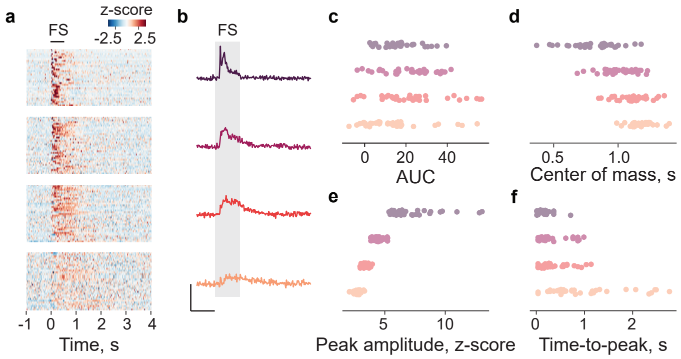

a
FS
z-score
-2.5
2.5
-1
0
1
2
3
4
Time, s
b
FS
c
0
20
40
AUC
d
0.5
1.0
Center of mass, s
e
5
10
Peak amplitude, z-score
f
0
1
2
Time-to-peak, s

Supplementary Fig. S2. Features of the Pavlovian discrimination task.

(**a**) Categorical heatmap depicting mice performance during conditioning session 1 and 2. (**b**) Time course of the average eye area changes across CS^+ and CS^- trials during habituation (left) and conditioning (session 1: center, session 2: right) (n_{mice} = 10). (**c**) Time course of the normalized eye closure for CS^+ and CS^- trials (bin = 3) during habituation and conditioning sessions. (**d**) Boxplots of the conditioned response amplitude for CS^+ and CS^- across habituation and conditioning sessions (n_{mice} = 10; Two Way ANOVA RM and Sidak's multiple comparison test, CS^+ vs CS^- $F_{(1,9)}$=87.66; H: p=0.99; C1: **p=0.022; C2: *p=0.018). (**e**) Averaged time course of the discrimination score (bin = 3) during habituation and conditioning sessions (n_{mice} = 10). (**F**) Boxplots of the discrimination score habituation and conditioning sessions (n_{mice} = 10; Two Way ANOVA RM; $F_{(1.378,\ 12.38)}$ = 4.31, *p=0.049).

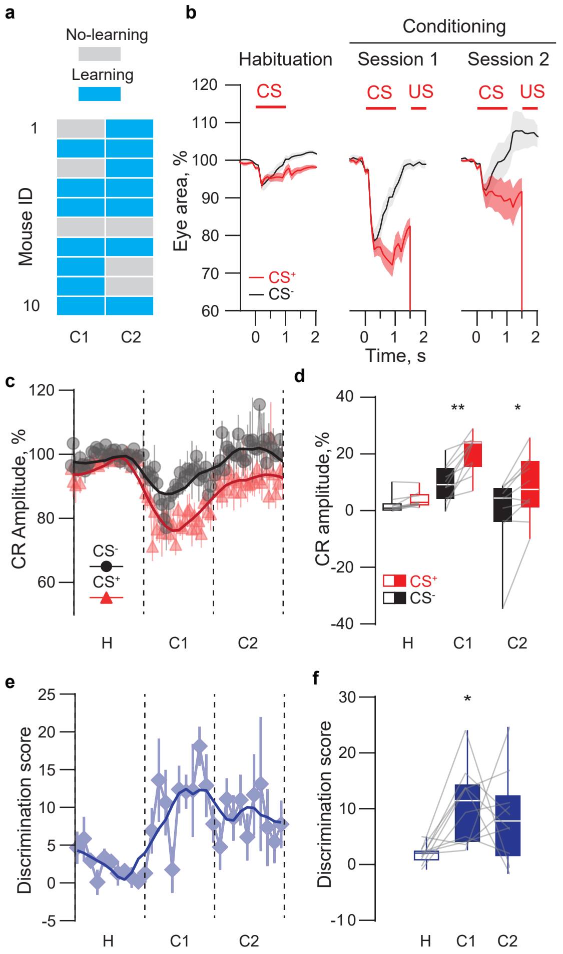

a
No-learning
Learning
1
Mouse ID
10
C1
C2
b
Conditioning
Habituation
Session 1
Session 2
CS
CS
US
CS
US
Eye area, %
CS+
CS-
Time, s
c
CR Amplitude, %
CS-
CS+
H
C1
C2
d
CR amplitude, %
**
*
CS+
CS-
H
C1
C2
e
Discrimination score
H
C1
C2
f
Discrimination score
*
H
C1
C2

Supplementary Fig. S3. Optical inhibition of LHb during the Pavlovian discrimination task.

(**a**) Schematic of the experimental setting for the infection of the AAV-Jaws in the LHb and example histological coronal section of the expression of Jaws in the LHb (left; scale bar: 200 µm) and schematic of the experimental protocol for the electrophysiological recordings of LHb neurons in anesthetized mice. (**b**) Time course of the average firing rate of the recorded LHb neurons during light delivery (n_{mice} = 3, $n_{neurons}$ = 15). (**c**) Boxplot of the average firing rate before, during and after red light presentation (n_{mice} = 3, $n_{neurons}$ = 15; Two Way ANOVA RM; $F_{(1.06,\ 14.78)}$ = 12.46, **p=0.003). (**d**) Example trace, raster plot and peristimulus time histogram of an example neuron inhibited by light presentation. (**e**) Fiber placement for all Jaws mice (left) and additional example pictures of Jaws expression in the experimental mice (right; scale bar: 200 µm). (**f**) Bar-graph representing the average relative arbitrary fluorescence measured in the LHb and neighboring regions (n_{mice} = 10, one-way ANOVA RM, $F_{(2.520,\ 22.68)}$ = 12.81, *p<0.0001) (**g**) Averaged time course of the discrimination score (bin = 3) during habituation and conditioning sessions for GFP (left) and Jaws (right) expressing mice (n_{mice} GFP = 7; n_{mice} Jaws = 10; habituation GFP vs Jaws: Kolmogorov-Smirnov test, D = 0.101, p=0.168; conditioning GFP vs Jaws: Kolmogorov-Smirnov test, D = 0.183, ***p<0.001).

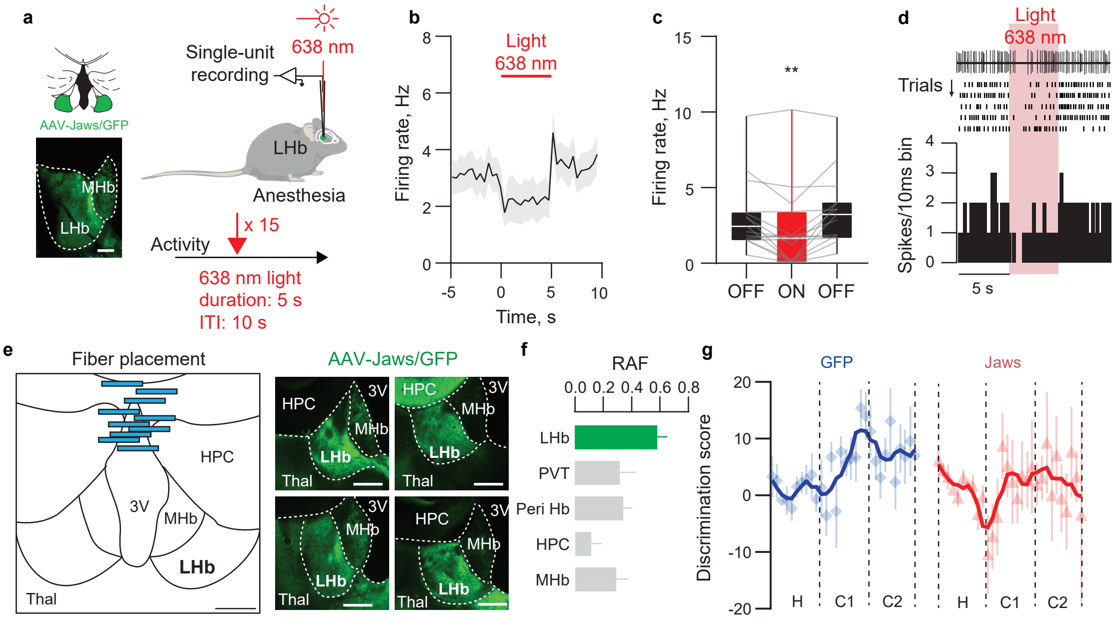

a
AAV-Jaws/GFP
MHb
LHb
Single-unit recording
638 nm
LHb
Anesthesia
x 15
Activity
638 nm light
duration: 5 s
ITI: 10 s
b
Light
638 nm
Firing rate, Hz
Time, s
c
**
Firing rate, Hz
OFF
ON
OFF
d
Light
638 nm
Trials
Spikes/10ms bin
5 s
e
Fiber placement
HPC
3V
MHb
LHb
Thal
AAV-Jaws/GFP
f
RAF
LHb
PVT
Peri Hb
HPC
MHb
g
GFP
Jaws
Discrimination score
H
C1
C2

Supplementary Fig. S4. Immunolabeling of glutamatergic and GABAergic neurons in the LHb.
(**a**) Representative confocal z-stack projections of LHb from mice infected with AAV-GCaMP6f, and relative quantification of GCaMP6f fluorescent cells co-localizing with EAAC1 (shown as % of positive cells out of GCaMP6f-positive total cells). (**b**) Same as A but for GAD67 immunoreactivity. (**c**) Representative confocal z-stack projections of EAAC1 and GAD67 immunoreactivity in the somatosensory cortex of the same mice (n_{mice}=4. Scale bar=30μm).

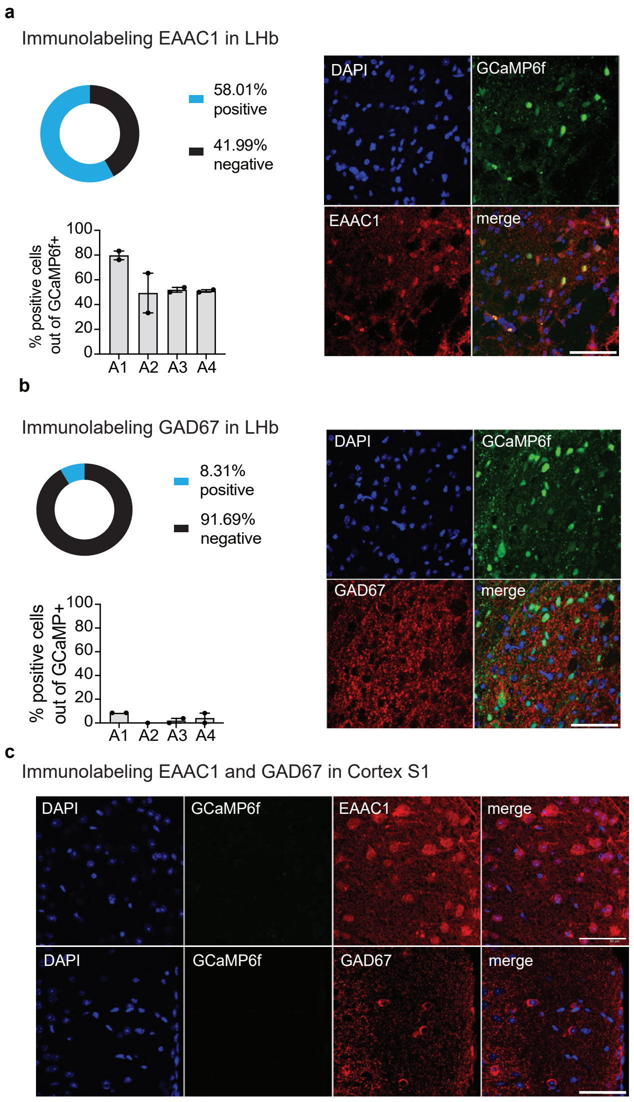
a
Immunolabeling EAAC1 in LHb
58.01% positive
41.99% negative
% positive cells out of GCaMP6f+
100
80
60
40
20
0
A1 A2 A3 A4
DAPI
GCaMP6f
EAAC1
merge
b
Immunolabeling GAD67 in LHb
8.31% positive
91.69% negative
% positive cells out of GCaMP+
100
80
60
40
20
0
A1 A2 A3 A4
DAPI
GCaMP6f
GAD67
merge
c
Immunolabeling EAAC1 and GAD67 in Cortex S1
DAPI
GCaMP6f
EAAC1
merge
DAPI
GCaMP6f
GAD67
merge

Supplementary Fig. S5. Functional and anatomical signatures of LHb neurons during conditioning.
(**a**) Example heatmap for a single mouse depicting time spent in the compartment paired with the CS+ auditory tone vs the not paired one. Boxplots and individual values reporting time in compartments (n_{mice} = 8; paired t-test; t_7 = 1.171, p = 0.280, paired t test). (**b**) Sankey-diagram representing neuronal responses to the US and CS^+ during conditioning (left) and bar graph representing the quantification of the percentage of neurons that are responding to both CS^+ and US or only US (right; X^2= 14.76, ***p<0.0001). (**c**) Scatter plot of the single neuronal response for CS^+ and US (n_{mice}=12, $n_{neurons}$=339; Pearson correlation, R^2 = 0.566, $F_{(1,337)}$ = 440.2, ***p < 0.0001). (**d**) Histological example sections of GRIN lens placement above the LHb (top) and respective field of view (FOV) average projection (bottom). (**e**) Anatomical reconstruction of excited and inhibited neuron putative location in horizontal plane and relative quantification of the medio-lateral spatial distribution (X^2= 66.56, ***p<0.0001).

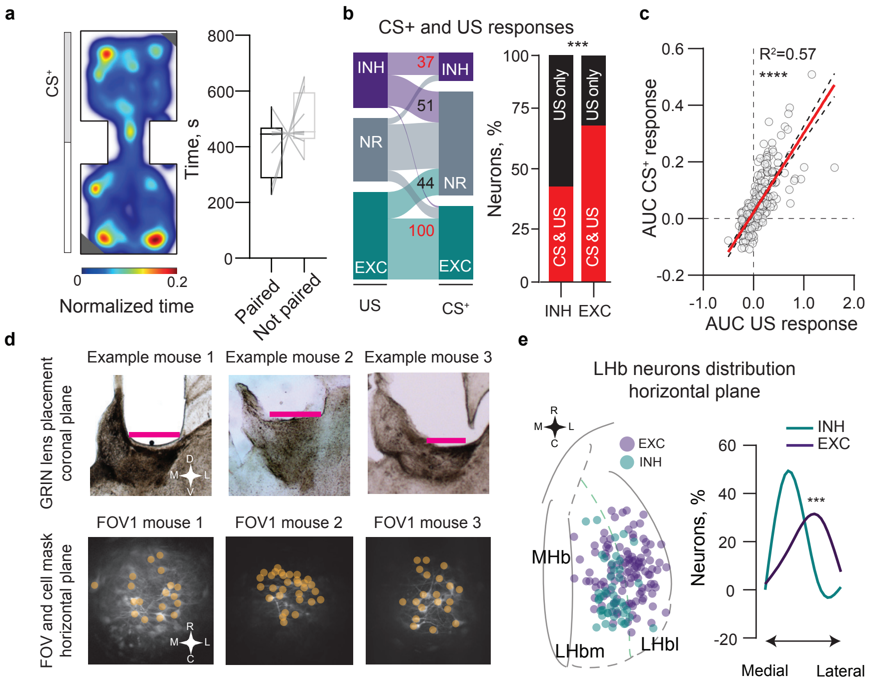
a
CS+
0
0.2
Normalized time
800
600
400
200
0
Time, s
Paired
Not paired
b
CS+ and US responses
INH
NR
EXC
37
51
44
100
US
CS+
100
75
50
25
0
Neurons, %
***
US only
CS & US
INH
EXC
c
R²=0.57
****
AUC CS+ response
AUC US response
0.6
0.4
0.2
0.0
-0.2
-1.0
0.0
1.0
2.0
d
Example mouse 1
Example mouse 2
Example mouse 3
GRIN lens placement coronal plane
FOV1 mouse 1
FOV1 mouse 2
FOV1 mouse 3
FOV and cell mask horizontal plane
e
LHb neurons distribution horizontal plane
EXC
INH
MHb
LHbm
LHbl
INH
EXC
60
40
20
0
-20
Neurons, %
***
Medial
Lateral

Supplementary Fig. S6. Potentiation of synaptic excitation in the LHb after learning.
(**a**) Schematic of the experimental setting for the behavioral training (left), training protocol for the two experimental groups and example of behavioral performance (center), and schematic representation of the location of the electrophysiological patch clamp recordings (right). (**b**) Example traces (top; scale bars: 50pA, 50 ms) and boxplots (bottom) of the AMPA/GABA ratio (n_{mice}: U = 2, P = 3, $n_{neurons}$: U = 9, P = 11; unpaired t-test, t_{18}= 3.53, **p=0.0024). (**c**) Example traces (top; scale bars: 50pA, 10ms) and boxplots (bottom) of the AMPA/NMDA ratio (n_{mice}: U = 3 P = 4, $n_{neurons}$: U = 5, P = 9; unpaired t-test, t_{12} = 2.323, *p=0.039). (**d**) Example traces (top; scale bars: 50 pA, 10 ms) and boxplots of the EPSC PPR (bottom; n_{mice}: U = 2, P = 2, $n_{neurons}$: U = 10, P = 11; unpaired t-test, t_{18} = 0.14, p=0.8941). (**e**) Example traces (top; scale bars: 250 ms, 20 pA), boxplots of the spontaneous EPSC frequency (bottom left; n_{mice}: U = 2, P = 3; $n_{neurons}$: U = 7, P = 9; unpaired t-test, t_{14} = 0.09, p=0.92) and boxplots of the spontaneous EPSC amplitude (bottom right; n_{mice}: U = 2, P = 3; $n_{neurons}$: U = 7, P = 9; unpaired t-test, t_{14} = 2.55, *p=0.023).

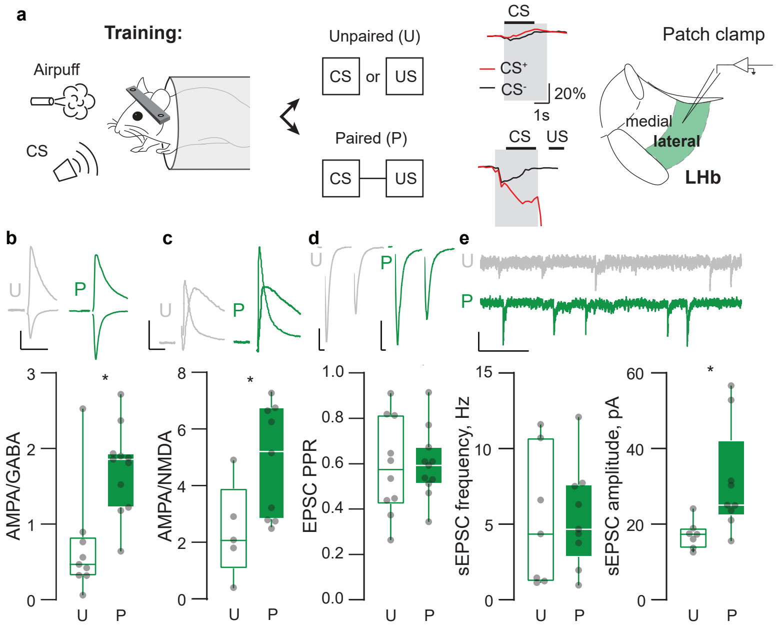

a
Training:
Airpuff
CS
Unpaired (U)
CS
or
US
Paired (P)
CS
US
CS
CS+
CS-
20%
1s
CS
US
Patch clamp
medial
lateral
LHb
b
U
P
AMPA/GABA
U
P
c
U
P
AMPA/NMDA
U
P
d
U
P
EPSC PPR
U
P
e
U
P
sEPSC frequency, Hz
U
P
sEPSC amplitude, pA
U
P

Supplementary Fig. S7. Analysis of neurotransmitter dynamics in the LHb.

(**a-d**) Left, fiber placement (left, scale bar: 200 μm); centre left, binary heatmap representing single mouse significant response to unpredicted airpuff and cue presentation during the habituation session (responses were considered significant when the average photometric responses was higher or lower than baseline for at least 150 ms); centre right, Time course of the maximum fluorescence (normalized to habituation) detected during CS+ and CS- presentation across training; right, boxplot depicting the maximum fluorescence detected during cue presentation normalized to the habituation session (Paired t-test. iGluSnFR: n_{mice} = 5; t_4 = 0.721, p=0.511. iGABASnFR2: n_{mice} = 3; t_2 = 0.010, p=0.993. GRAB5-HT2h: n_{mice} = 3; t_2 = 0.81, p=0.503. GRAB-ACh3.0: n_{mice} = 6; t_5 = 3.14, *p=0.026).

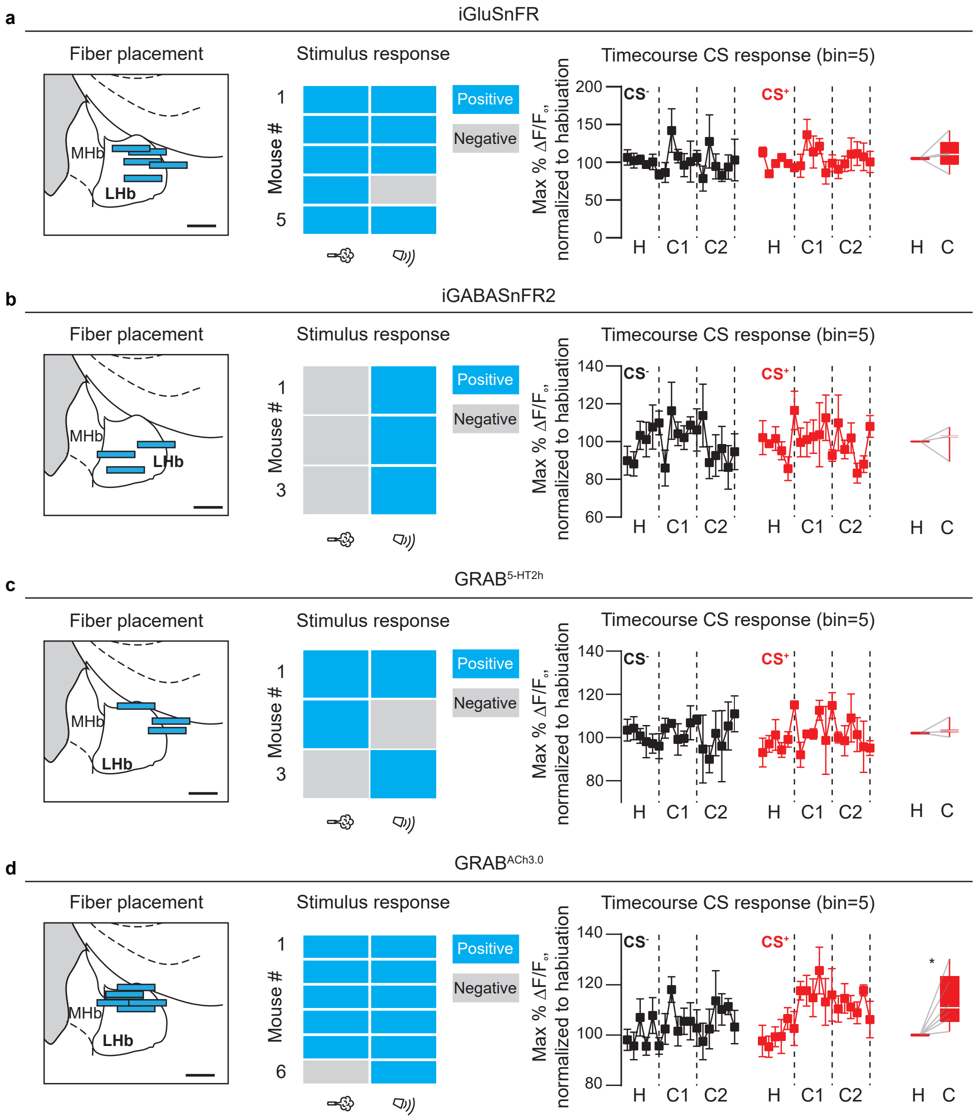
a
iGluSnFR
Fiber placement
MHb
LHb
Stimulus response
Mouse #
1
5
Positive
Negative
Timecourse CS response (bin=5)
Max % ΔF/F$_0$, normalized to habiuation
200
150
100
50
0
CS-
CS+
H
C1
C2
H
C
b
iGABASnFR2
Fiber placement
MHb
LHb
Stimulus response
Mouse #
1
3
Positive
Negative
Timecourse CS response (bin=5)
Max % ΔF/F$_0$, normalized to habiuation
140
120
100
80
60
CS-
CS+
H
C1
C2
H
C
c
GRAB^{5-HT2h}
Fiber placement
MHb
LHb
Stimulus response
Mouse #
1
3
Positive
Negative
Timecourse CS response (bin=5)
Max % ΔF/F$_0$, normalized to habiuation
140
120
100
80
CS-
CS+
H
C1
C2
H
C
d
GRAB$^{ACh3.0}$
Fiber placement
MHb
LHb
Stimulus response
Mouse #
1
6
Positive
Negative
Timecourse CS response (bin=5)
Max % ΔF/F$_0$, normalized to habiuation
140
120
100
80
CS-
CS+
H
C1
C2
*
H
C
